# A Wearable Device for Breathing Frequency Monitoring: A Pilot Study on Patients with Muscular Dystrophy

**DOI:** 10.3390/s20185346

**Published:** 2020-09-18

**Authors:** Ambra Cesareo, Santa Aurelia Nido, Emilia Biffi, Sandra Gandossini, Maria Grazia D’Angelo, Andrea Aliverti

**Affiliations:** 1 Scientific Institute, IRCCS “E. Medea”, Bioengineering Lab, Bosisio Parini, 23842 Lecco, Italy; ambra.cesareo@polimi.it (A.C.); emilia.biffi@lanostrafamiglia.it (E.B.); 2 Dipartimento di Elettronica, Informazione e Bioingegneria, Politecnico di Milano, 20133 Milano, Italy; santaaurelia.nido@mail.polimi.it; 3 Scientific Institute, IRCCS “E. Medea”, Department of Neurorehabilitation, Neuromuscular Unit, Bosisio Parini, 23842 Lecco, Italy; sandra.gandossini@libero.it (S.G.); grazia.dangelo@lanostrafamiglia.it (M.G.D.)

**Keywords:** breathing monitoring, breathing rate variation, wearable IMUs, neuromuscular patients

## Abstract

Patients at risk of developing respiratory dysfunctions, such as patients with severe forms of muscular dystrophy, need a careful respiratory assessment, and periodic follow-up visits to monitor the progression of the disease. In these patients, at-home continuous monitoring of respiratory activity patterns could provide additional understanding about disease progression, allowing prompt clinical intervention. The core aim of the present study is thus to investigate the feasibility of using an innovative wearable device for respiratory monitoring, particularly breathing frequency variation assessment, in patients with muscular dystrophy. A comparison of measurements of breathing frequency with gold standard methods showed that the device based on the inertial measurement units (IMU-based device) provided optimal results in terms of accuracy errors, correlation, and agreement. Participants positively evaluated the device for ease of use, comfort, usability, and wearability. Moreover, preliminary results confirmed that breathing frequency is a valuable breathing parameter to monitor, at the clinic and at home, because it strongly correlates with the main indexes of respiratory function.

## 1. Introduction

In the severe forms of muscular dystrophy, such as Duchenne Muscular Dystrophy (DMD), respiratory failure is still the principal cause of death, followed by cardiomyopathy. Muscle weakness, ineffective coughing, and reduced ventilation often leads to pneumonia, atelectasis, and respiratory insufficiency during sleep and while awake [1]. Pulmonary involvement is observed also in patients with some form of limb girdle muscular dystrophy (LGMD) and may occur early in the disease [2,3,4]. Although no therapy is available, new wide-ranging and structured therapeutic approaches with increased attention to respiratory care help improve MD patients’ quality of life and life expectancy [5,6]. Periodic measurement of respiratory function and respiratory muscle strength allow the clinician to predict when to introduce assisted coughing and ventilation. Recommended respiratory evaluation includes measurement of oxyhemoglobin saturation, spirometric parameters, maximum inspiratory and expiratory pressures, and peak cough flow once or twice per year. These patients thus need a careful respiratory assessment, and periodic follow-up visits to monitor the progression of the disease are strongly suggested. Nevertheless, the optimal frequency of follow up is not known. In fact, most patients with muscular dystrophy do not realize that they have lost respiratory muscle strength and cough effectiveness until a respiratory viral infection leads to pneumonia. For these reasons, continuous monitoring of respiratory activity and breathing pattern between consecutive follow-up visits could provide additional understanding about disease progression, in addition to traditional, intermittent, cardiopulmonary evaluations, allowing prompt clinical intervention and anticipation of respiratory dysfunction. Moreover, the identification of early markers of respiratory dysfunction indexes may also support the creation of personalized plans of sequential follow-up, helping ameliorate the quality of life of dystrophic patients.

As widely documented in the literature, breathing frequency is an important variable of breathing and ventilatory patterns. An increased respiratory rate represents the most sensitive indicator of increasing respiratory difficulty [7]. Thus, respiratory rate is one of the vital signs that is primarily assessed on hospital admission. Nevertheless, importance of breathing rate goes beyond diagnosis. It allows discrimination between stable and at-risk patients [8], and can be used to predict potentially serious clinical events [9,10], in addition to monitoring the progression of illness [11,12,13]. For this reason, the importance is evident of breathing rate monitoring in clinical setting and after discharge, especially for those patients who are at high risk of developing cardio-respiratory dysfunctions, such as patients suffering from neuromuscular diseases and respiratory muscle weakness. Monitoring breathing frequency could be helpful to predict acute exacerbations or to assess spontaneous breathing trials during weaning from mechanical ventilation after intubation.

Continuous measurement of respiratory rate can be achieved by non-intrusive wearable devices. This consists of deriving a respiratory-related signal by detecting the motion of the thoraco-abdominal surface by inductive [14], resistive [15,16,17], or capacitive sensors [18]. More recently, smart textiles embedding fiber optic sensors, namely fiber Bragg grating (FBG) sensors positioned at different body locations, have also been proposed for respiratory monitoring [19]. An emerging approach is to derive breathing signal, and related parameters, by measuring chest wall breathing motions using small inertial sensors mounted on the external surface of the chest or abdomen. This approach is highly promising because it allows long recordings, without the need to increase dimensions and costs, or the necessity to change the habits of the patients. Many studies in the literature demonstrated the feasibility of systems based on mono- or tri-axial accelerometers to measure breathing frequency in healthy subjects in different positions and their ability to distinguish between different kinds of respiratory patterns [20,21,22,23,24,25].

In previous works, our group presented a device and a method based on magnetic-inertial measurement units aimed at monitoring breathing temporal parameters for prolonged periods, also providing preliminary validation in healthy adults [26,27,28]. Preliminary tests of the analysis method were also made in semi-static (posture changes) and dynamic (walking, light exercises) conditions, and provided encouraging results [28]. The next step involves the testing of the proposed device and processing algorithm on the target clinical population, both under static conditions and during daily activities.

The main objective of the present study is thus to investigate the feasibility of using an innovative wearable device for respiratory monitoring, especially breathing frequency variation assessment, in patients with muscular dystrophy. Specifically, we wanted (1) to assess the ability of the device to accurately estimate breathing parameters in patients presenting shallow breathing, in static condition; (2) verify the feasibility of using the device for long periods during daily life activities; (3) investigate usability and acceptability; and (4) preliminarily evaluate the possibility of using breathing frequency continuously assessed during daily activities as an additional marker of respiratory dysfunction.

## 2. Materials and Methods

### 2.1. Device Description

**Figure 1:**
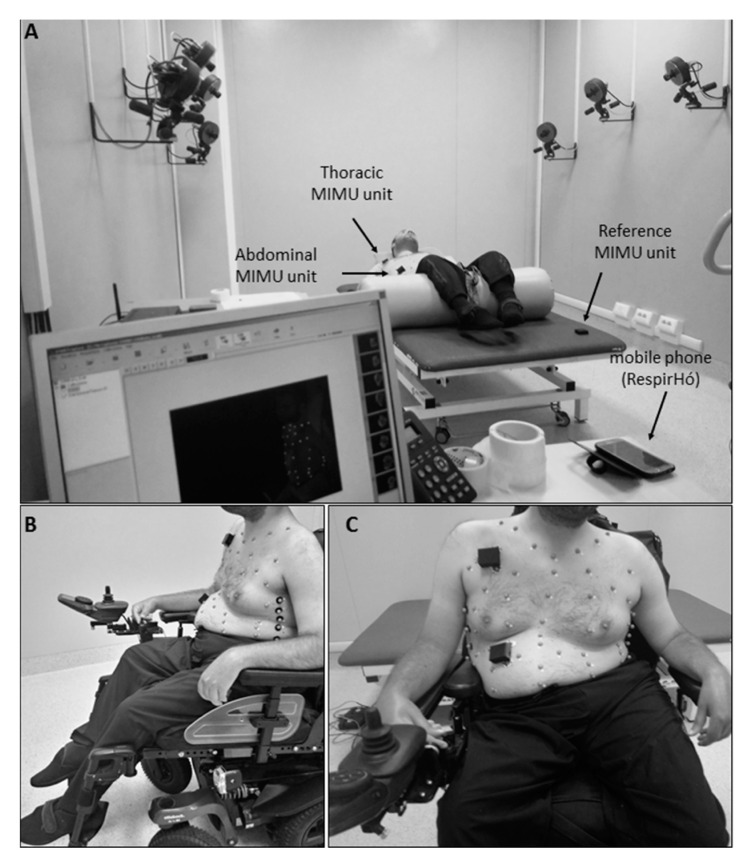
Experimental setup in static conditions. (**A**) Setup for acquisitions in supine position, with a view of the Optoelectronic Plethysmography laboratory. (**B**,**C**) Setup for acquisitions in seated position, lateral and frontal view, respectively.

The system used in the present paper is a wearable, unobtrusive inertial-sensor-based device for long-term breathing pattern monitoring, including during daily life activities. It consists of three inertial measurement units (IMU) (3-axis accelerometer, 3-axis gyroscope, 3-axis magnetometer), positioned on the patient’s abdomen and thorax (see Figure 1C), and on a body area integral with thorax but not affected by respiratory movements. The peripheral units, placed on thorax and abdomen, are used to record orientation changes during respiratory movements. The third unit is a central reference unit (hereafter CRU) that receives data from the other two units, save them on an SD card, and communicate via Bluetooth Low Energy (BLE) with a smartphone/tablet/PC. Moreover, this unit detects only non-respiratory movement, representing not only a pure source of “noise” that must be removed from the thoracic and abdominal signals, but also a pure source of additional information regarding the state of activity of the subject. A more detailed description is provided in [27,28]. The measurements provided by the IMU sensor are used by the microcontroller to calculate a quaternion, which represents orientations and rotations of the device units in three dimensions. An extensive description of the device firmware is provided in [26,27].

The CRU receives blocks of data from the two peripheral units and from its onboard sensor, according to a specific communication protocol. In particular, the BLE module on the CRU connects cyclically (using 5-second windows) to each unit, and receives and saves on the SD card a block of data, corresponding to the quaternion components evaluated in the previous 15 seconds. According to this communication protocol, it is necessary to re-synchronize the data coming from the three units as they are delayed by 5 seconds from each other. Every 3 minutes the data saved on the SD card, containing the data recorded by the 3 units, are sent to the smartphone, which saves the data in a .txt file named with the date and time in which the acquisition started. These operations are performed in about 45 s, during which the BLE of the central unit is connected to the smartphone and therefore does not receive the data recorded by the peripheral units. At the end of this process, the 3 units are restored, and the process described above restarts until the units are turned off. Thus, the device works as an acquisition platform to record data in blocks of 3 m spaced by 45-second periods.

### 2.2. Analysis Algorithm

The analysis needed to compute the respiratory parameters from the data collected by the device was performed offline using MATLAB. For each trial, mean values of fB, TI, and TE were extracted from the tracings obtained using the IMU-based device by applying the analysis algorithm proposed by Cesareo et al. [27], and using a reduction method based on principal components analysis (PCA-fusion). As a first step, this algorithm computes the quaternions that represent the orientation changes of (1) the abdominal unit with respect to the CRU unit and (2) thoracic unit with respect to the CRU unit, to remove non-respiratory movements recorded from CRU. Then, to maximize respiratory information, principal component analysis is applied to the four quaternion components [q0 q1 q2 q3] of each quaternion (thoracic and abdominal) and the first principal component is selected and used for further analysis. For each signal (thoracic and abdominal) the power spectral density (PSD) is computed by applying Welch’s method (window: 300 samples, overlap: 50 samples, DFT length: 512 points) and the frequency associated with breathing (fpeak) is determined. According to this preliminary spectral analysis, a band-pass filter (first-order IIR Butterworth filter) centered on fpeak frequency was applied to the signals, and parametric tuning was performed by selecting a set of parameters to optimize subsequent analysis phases. Signals were then smoothed using a third-order Savitzky–Golay FIR filter, and maxima and minima points representing beginning and end of inspiratory and expiratory phases, respectively, were detected. Finally, on a breath-by-breath basis, inspiratory time (TI), expiratory time (TE), and total time (TTOT) were computed and “instantaneous” breathing frequency expressed in breaths/minute was derived as 60/(TTOT). Finally, we considered the average value of each parameter (TI, TE, TTOT, fB) over each trial.

### 2.3. Clinical Protocol

The clinical protocol described in this pilot study was approved by the Ethics Committee of the Scientific Institute IRCCS Eugenio Medea, located in Bosisio Parini, Italy, in accordance with the declaration of Helsinki and by the Italian Ministry of Health as a clinical investigation involving medical devices not bearing the CE mark.

#### 2.3.1. Participants

Among the neuromuscular patients attending the Scientific Institute IRCCS “E. Medea” for periodic clinical assessment, only those affected by Duchenne Muscular Dystrophy or Limb-Girdle Muscular Dystrophy–type R (previously symbolized as LGMD2) were enrolled in the study. These patients are at high risk of developing respiratory dysfunctions. Diagnosis of DMD and LGMD2 was based on clinical, genetic, and/or histological data [6,29,30]. Inclusion criteria were, other than documented DMD or LGMD2, loss of independent ambulation (wheelchair-bound patients), and ability to understand and follow test instructions and to report pain and discomfort. Exclusion criteria were: presence of metal implants and cardiac pacemakers, relevant concomitant comorbidities (e.g., epilepsy), behavioral and/or psychiatric disorders (e.g., emotional problems, anxiety, panic attacks).

For all of the participants, clinical information, including use of non-invasive mechanical ventilation, years of use of cough assistive devices, corticosteroids, cardiac function, severity of scoliosis, presence of spinal fusion, nutritional status and use of percutaneous endoscopic gastrostomy (PEG), was recorded.

All participants and their legal representatives were informed about the study and signed a consent statement.

#### 2.3.2. Respiratory Function Assessment

Respiratory function was evaluated by assessing spirometry, pulse-oximetry, maximal respiratory pressures, and cough peak flow (CPF), according to the guidelines for respiratory muscles testing [31,32,33].

Pulmonary Function Tests: The following spirometric (Vmax series 22; SensorMedics, Yorba Linda, CA, USA) pulmonary function parameters were recorded: forced vital capacity (FVC), forced expiratory volume in 1 second (FEV1), forced expiratory flow at 25–75% of FVC (FEF25–75%), forced expiratory flow at 50% of FVC (FEF50%), and peak expiratory flow (PEF). Moreover, subdivisions of lung volumes (functional residual capacity (FRC), residual volume (RV), and total lung capacity (TLC)), were obtained using the nitrogen washout technique. Nocturnal oxygen saturation (SpO2) was assessed by using a digital pulse-oximeter (Nonin, 8500 digital pulse oximeter Quitman, TX).

Respiratory Muscle Strength: measurements of maximal inspiratory and expiratory pressures (MIP and MEP) were obtained at the mouth (MicroRPM; Micro Medical Ltd., Rochester, England) starting respectively from TLC and RV and maintaining the effort for at least one second. The highest values of MEP and MIP obtained from two or more tries were considered.

Cough effectiveness: Effectiveness of coughing was assessed by measuring the maximum unassisted cough peak flow (CPF) using a portable peak flowmeter (Vitalograph, Ennis, Ireland). Patients were asked to cough with maximal strength two times and then the highest value from the two trials was considered.

#### 2.3.3. Experimental Procedures

##### Phase A: Laboratory Validation

To assess the efficacy of the IMU-based device in correctly estimating breathing parameters in neuromuscular patients, chest wall movements during breathing were simultaneously recorded using the IMU-based device and gold standard method in static conditions, and in particular, in supine and seated positions (Figure 1). The reference method used in this study was Optoelectronic Plethysmography (OEP), which has been widely validated in different conditions and positions. This technique proved to have intra-rater and inter-rater reliability and discrepancies in tidal volume measurements were always <5% [34,35,36,37,38,39,40]. The decision to use OEP as reference method is mainly due to the fact that it is based on similar functioning principles of the IMU-based device; in fact, it measures chest wall movements related to breathing to assess ventilatory and breathing patterns; rather than using IMUs, OEP relies on motion capture principles. The system used in the present study (BTS-OEP System, BTS Bioengineering) has eight infrared video cameras (sampling rate: 60 Hz) used to capture the light reflected by retro-reflective markers positioned on the chest wall at specific anatomic points. The system is able to compute the 3D coordinates of each marker if the same marker is seen by at least two cameras simultaneously (stereophotogrammetry). From the 3D coordinates of the markers, it is possible to approximate the chest wall surface and then to compute the volume enclosed by this surface (Gauss’s theorem). Variations of the enclosed volume can be, with optimal approximation, associated with the respiratory activity. This means that studying the chest wall volume variations allows us to assess the ventilatory and breathing patterns. Moreover, by modelling the chest wall as being composed of rib cage and abdomen, it is possible to investigate the contribution of both the compartments to total chest wall volume. This is an interesting advantage for the validation of the IMU-based device, because it allows the data recorded with the IMU-based device to be compared with measurements obtained using the reference method (OEP) at the level of the two compartments of interest (thorax and abdomen). This would not be possible with other standard methods such as spirometry.

For acquisition in the supine position, the subjects were prepared according to a 52-marker protocol [41,42]. The peripheral IMU units of the device were placed on the thorax and on the abdomen, while the reference IMU unit was placed on the bed. For measurement in seated position, the same 52-marker protocol was used for patients unable to sit without back support, for whom acquisition was performed in their wheelchair; the reference IMU-unit was placed on the seventh cervical vertebrae (C7) or on the back of the wheelchair. Patients who were able to maintain a static trunk position performed the acquisition seated on the bed, using an 89-marker configuration [36,43] and applying the reference IMU-unit on the coccyx.

The acquisition protocol included two quiet breathing (QB) trials of 3 minutes including a slow vital capacity maneuver (SVC) at the begin of the trial. QB means breathing quietly in a natural way without speaking. The SVC maneuver is a maneuver in which the subjects must perform a maximal inspiration followed by a maximal expiration, and is generally clearly recognizable compared to quiet breathing inside a breathing tracing. For this reason, it was included in the trial to provide reference timing to align the OEP signal and IMU-based signals during data analysis.

##### Phase B: Daily Use Assessment

The second part of the protocol was aimed at investigating the feasibility of using the device for prolonged periods of time, during daily activities, which included nutrition, sleep, wheelchair movement, speech, and activities planned for the day hospital. Subjects and their caregivers were trained to autonomously use the device and were helped for the initial placing of the IMU units. They received instructions about the possibility of interrupting the acquisition when needed and restarting it again, provided that any relevant event was properly reported in a diary. Possible causes of acquisition interruption could be clinical examinations and personal hygiene routine. Furthermore, they were asked to record in the diary the activities that they carried out during the day with relative times. For each patient the device was worn for a variable period of time and for different periods of the day based on their personal commitments.

At the end of the period of independent and autonomous use, subjects were asked to reply to some evaluation questionnaires, to collect feedback about usability (System Usability Scale, [44,45,46,47]) acceptance, and wearability of the device (ad-hoc questionnaire, see Appendix A).

### 2.4. Measurements and Statistical Analysis

#### 2.4.1. Validation in Static Conditions

For each quiet breathing trial, mean values of fB, TI, and TE were extracted from the tracings (abdomen and thorax). The same parameters were extracted from tracings obtained using OEP, on the abdominal and thoracic compartments. For each trial, a period of at least 30 seconds manually selected by an operator was considered to compute the mean values. For each parameter, measurements obtained using the IMU-based device were compared to those obtained using OEP. The comparison between the two methods was performed considering accuracy, correlation, and agreement. Regarding accuracy, the absolute (Equation (1)) and relative (Equation (2)) errors of estimation were computed:

(1) Absolute Error (E)=|Device−OEP|

(2) $$Relative\;.Error\;\left(E\%\right)\frac{\left|Device-OEP\right|}{OEP}*100$$

Median and interquartile range (75th percentile–25th percentile) were computed for E and E% for all subjects and all trials, for both thoracic and abdominal compartments, considering seated and supine positions. Linear regression analysis and correlation analysis were performed for each parameter (fB, TI, TE,), comparing measurements obtained with the IMU-based device with those from OEP. Pearson’s product-moment correlation, rP, was used for normal distributions, while Spearman’s rank-order correlation, rS, was used when data were not normally distributed. To assess the normality of data we used the Shapiro–Wilk normality test. The agreement with the refence method was assessed by using Bland–Altman analysis, which requires the differences of the two paired measurements (Device − OEP) to be plotted against the mean of the two measurements [48,49,50]. Heteroscedasticity of data was investigated as proposed by Brehm et al. [51] to assess the presence of proportional biases and/or the correlation between differences and mean values. To do so, Kendall’s tau (τ) correlation between the absolute differences and the corresponding means was computed and, when a positive significant correlation (τ > 0.1 and *p*-value < 0.05) emerged, data were denoted heteroscedastic. For homoscedastic data, mean of the differences (d) and limits of agreement (LOA: from −1.96 × SD to +1.96 × SD) were calculated. When heteroscedasticity was present, the approach based on the construction of V-shaped limits was used: the mean bias (d) is replaced by the regression line of the points (ordinary least squares (OLS) best fit) and the fixed LOAs, characterized by constant standard deviation, are replaced by V-shaped confidence limits (upper: UCL and lower: LCL), around the regression line of the differences [52,53].

#### 2.4.2. Long-Term Breathing Pattern Monitoring (Daily Use)

Data recorded during Phase B of the protocol were used at first to obtain insights on duration, data loss, and efficiency of the device. Time of use of the device, and voluntary (participants or caregivers intentionally turned off the device) and unexpected (due to communications problems) interruptions of the acquisitions were recorded. As a consequence of these interruptions, the length of time for which the device collected data, in some cases, was less than the time frame in which the patient used the device autonomously. Moreover, loss of data due to synchronization procedures and BLE transmission may have occurred and been described. The following parameters characterizing duration, data loss, and efficiency were computed:

- Autonomous use time: duration of time during which the patient autonomously used the device.
- Intrinsic data waste: equal to the difference between the expected duration of the acquisition and the actual duration of the recorded data. The latter was obtained as the number of recorded files multiplied by the expected duration of each file (155 s). The intrinsic waste of data is due to limitations of the transmission protocol that requires: (1) resynchronization of data sent by the abdominal, thoracic, and reference units with a consequent waste of initial and final data for each block; and (2) sending of each 3-min block data from the reference unit to the smartphone, which is an operation requiring about 45 s during which the system cannot acquire data.
- Efficiency in terms of data analysis: expressed as the number of files that can be actually analyzed (at least 30 s of consecutive data must be available to compute the PSD and correctly execute the analysis algorithm), with respect to the number of total files recorded.
- Number of unexpected interruptions (N. of unexpected interruption).

In addition to this analysis, each acquisition block recorded during Phase B was analyzed by applying the same processing algorithm used for static conditions, to extract information about breathing frequency. An operator was needed to supervise the analysis ensuring that reliable sequences of breaths were evaluated. A mean value of breathing frequency over the selected breaths (at least 30 consecutive seconds) was subsequently computed for each acquisition block and each compartment (abdomen, thorax) obtaining a plot of breathing frequency variations over time. Ranges of breathing frequencies (mean ± SD) obtained from OEP during the tests in static conditions, for supine and seated positions, are also reported as a reference. The activity diary, together with raw quaternion signals from IMU units, was used to discriminate static from dynamic periods and to track the activities carried out during the recording.

#### 2.4.3. Usability and Acceptability

Regarding the evaluation of the System Usability Scale (SUS) and the ad-hoc questionnaire results, the items were presented as 5-point scales numbered from 1 (“Strongly disagree”) to 5 (“Strongly agree”). Each item’s score contribution ranged from 0 to 4: for positively-phrased items (such as “I think that I would like to use this system frequently”), the score contribution was obtained as the scale position minus 1. For negatively-worded items (such as “I found the system unnecessarily complex”), the score was obtained as 5 minus the scale position. The overall score for both of the scales was obtained by multiplying the sum of the item score contributions by 2.5. Thus, scores ranged from 0 to 100 in 2.5-point increments, with higher values meaning higher perceived usability of the system. For both questionnaires the average value ± SD obtained from all of the questionnaires submitted to the subjects were reported. Moreover, radar plots reporting the average scores for each item of the questionnaires were described to provide a detailed analysis of usability and acceptance.

#### 2.4.4. Breathing Frequency: A Potential Marker of Respiratory Dysfunction

To preliminarily investigate the possibility of using breathing frequency as a marker of respiratory dysfunction, boxplots representing breathing frequency variations during long-term breathing pattern monitoring (Phase B) were created for each patient, considering different conditions, such as using noninvasive mechanical ventilation (NIV) or not, and day/night. The objective was to compare breathing frequency distributions in participants with muscular dystrophy to normal physiological ranges in adolescents and adults.

Moreover, correlation and regression analyses were performed between the median values of the estimated respiratory frequencies obtained during Phase B (no NIV, day hours) and the most common indexes of respiratory function (PEF%, FVC%, and PCF), measured during the respiratory function assessment.

## 3. Results

### 3.1. Participants

Fifteen male neuromuscular subjects (13 DMD and 2 LGMD) were enrolled. Table 1 shows anthropometric and clinical characteristics of the subjects, reported as mean ± standard deviation (SD). The dataset was divided into two groups: patients with DMD and patients with LGMD.

All of the DMD subjects were wheelchair bound with an average loss of ambulation age of 9.98 ± 2.07 years; one patient at the time of the test had poor ambulation ability; LGMD patients were wheelchair bound and lost ambulation capability at 48 and 42 years old, respectively. All DMD subjects presented scoliosis, with different degrees of severity, and two underwent posterior spinal fusion. Seven subjects had been previously treated with steroids for at least 2 years and four subjects were receiving steroid treatment at the time of evaluation. Subjects presenting heart dysfunction (identified mainly with a left ventricle ejection fraction lower than 50%) were receiving b-blockers, ACE (Angiotensin-converting enzyme) inhibitors, or both treatments at the time of the study. Eleven subjects were using noninvasive mechanical ventilation (NIV) and began to use it, on average, at 22.00 ± 6.90 years of age. Two patients also used NIV during daytime, for a total amount of time of 18/22 hours per day. Three patients also used NIV during daytime for a few hours (2–4). Ten subjects presented a good nutritional condition (BMI > 18 and BMI < 25), four subjects were affected by pathological thinness (BMI < 18) with swallowing disturbances, and one subject presented a BMI > 25. None of the patients were using PEG.

### 3.2. Respiratory Function

Spirometric parameters (FVC, FEV1, FEF25-75, FEF50, and % with respect to the predicted values) and lung volumes (TLC, RV and FRC, and % values) are reported as mean ± standard deviation for all of the participants in Table 2. Four DMD subjects did not perform the spirometry test, three of which due to severe facial muscular weakness or macroglossia.

Regarding respiratory muscle strength assessment, mean ± SD MIP and MEP were 35.57 ± 28.00 cmH_2_O and 35.29 ± 30.39 cmH_2_O, respectively; 6 of the 14 evaluated patients presented both MIP and MEP values <20 cmH_2_O. Mean PCF ± SD was 173.33 ± 107.96 L/min, with 4 of the 10 evaluated patients presenting ineffective cough (PCF < 160 L/min). Mean ± SD oxygen saturation SpO_2_ at night was 95.81 ± 1.61, with only six subjects presenting mild signs of nocturnal oxygen desaturations (spending more than 10% of the nighttime with SpO_2_ < 95%).

These results fit with the clinical picture characterized by restrictive lung pattern, respiratory muscle weakness (decreased MIP and MEP), and ineffective cough.

### 3.3. Validation in Static Conditions

Table 3 shows absolute and relative estimation errors relative to breathing frequency, and inspiratory and expiratory times.

**Figure 2:**
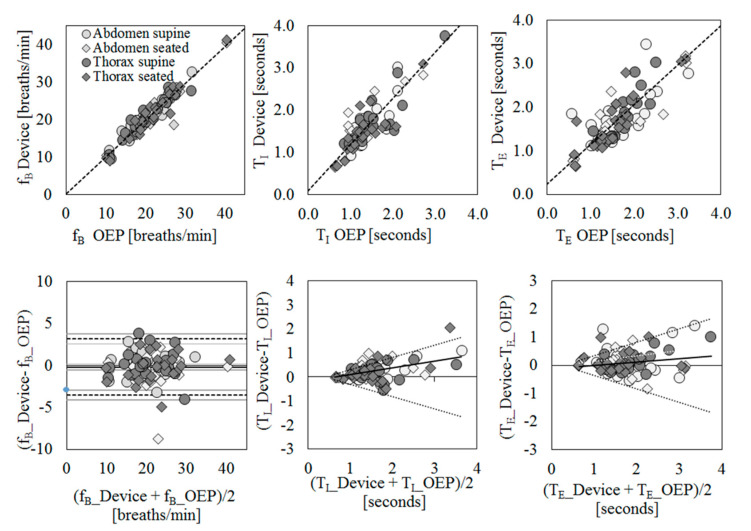
Agreement between IMU-based device and Optoelectronic Plethysmography (OEP) for the estimation of breathing frequency (f**_B_**, first column), inspiratory time (T_I_, second column), and expiratory time (T**_E_**, third column) using regression (first row) and Bland–Altman (second row) analysis. For regression analysis, scatter plots of measurements of the IMU-based device vs. OEP are shown. Regression equations: f**_B_**_Device = 0.98* f**_B_**_OEP + 0.22; T_I___Device = 1.09* T_I___OEP + 0.11; T**_E_**__Device = 0.91* T**_E_**_OEP + 0.23. For agreement analysis, Bland–Altman plots are shown, where the differences (IMU-based device-OEP) are plotted against the mean of the two measurements. The breathing frequency plot shows the mean of the differences (–––), limits of agreement (- - -) from d − 1.96 s to d + 1.96 s, and representation of 95% confidence interval limits for mean and agreement limits (grey bands). For heteroscedastic data (T_I_ and T**_E_**), the proportional bias (—) is represented by the ordinary least squares (OLS) line of best fit for the difference of mean values; V-shaped upper and lower 95% confidence limits (····) are calculated according to Bland

Scatter plots of measurement obtained using the IMU-based device vs. OEP and Bland–Altman plots are reported for each parameter (Figure 2), considering the data obtained from the thoracic and abdominal compartments, both in supine and in seated positions, for all participants as a unique dataset.

Correlation coefficients between the measurements obtained with the IMU-based device and OEP yielded statistically significant results (n = 98; fB: rS = 0.942 *p* < 0.001; TI: rS = 0.778, *p* < 0.001; TE: rS = 0.797, *p* < 0.001). Regarding the Bland–Altman analysis, only the fB dataset was found to be homoscedastic, i.e., no significant correlation emerged between differences and mean values (Kendall’s correlation; fB: τ = −0.088, *p* = 0.205; TI: τ = 0.399, *p* = 0.000, TE: τ = 0.285, *p* = 0.000), thus a “classic” Bland–Altman plot was drawn for fB, including computation of mean of differences between the IMU-based device and OEP measurements (fixed bias: d), and upper and lower limits of agreement (d ± 1.96 × SD), together with their 95% confidence intervals (CI). For fB, the mean of difference was −0.183 (95% CI from −0.526 to 0.159: not significant fixed bias); breaths/min and LOAs ranged from −3.531 (95% CI from −4.124 to −2.938) breaths/min to 3.164 (95% CI from 2.570 to 3.757) breaths/min. Only three points out of 98 were outside the range of agreement (3.06%).

With regard to respiratory times, both datasets were found to be heteroskedastic and worse results were obtained (TI: proportional bias: y = 0.29x − 0.19, UCL: y = 0.51x − 0.20, LCL: y = −0.51x + 0.20; TE: proportional bias: y = 0.12x − 0.11, UCL: y = 0.47x − 0.11, LCL: y = −0.47x + 0.11.

### 3.4. Long-Term Breathing Pattern Monitoring (Daily Use)

With the exception of Subject #1, other participants participated in Phase B of the protocol wearing the device during their daily activities and/or sleep. The participants used the device for a mean time of 09:37 h. Five of these were in-patients and used the device mainly during night, thus allowing an overnight recording; the other patients were out-patients and/or used the device for a few hours during the day. Overall, 9 of the 15 patients used the device for more than 6 hours. Twelve unexpected interruptions to acquisition occurred, due to the BLE transmission protocol, and a value of 31% of intrinsic data waste was recorded due to limitations of the transmission protocol (i.e., synchronization of the three units, time needed to send data to the smartphone). Nevertheless, the mean efficiency in terms of data analysis was 85.18 ± 20.98; this means that a mean breathing frequency was extracted from about 85% of the recorded files.

**Figure 3:**
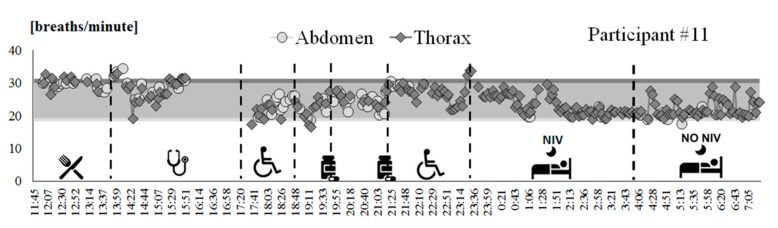
Breathing frequency variation over time recorded by using the IMU-based device on participant #11, from 11:45 a.m. to 7:15 a.m. of the next day. Each point represents the mean value computed over a 3-minute bock, for thoracic (dark grey diamonds) and abdominal (light grey circles) signals. Ranges of breathing frequencies recorded during static acquisitions using OEP for supine (light grey band) and seated (dark grey band) positions are reported as reference. The activities performed by the subject are represented on the bottom.

A tracing of breathing frequency variation over time was obtained for all of the participants that participated in Phase B of the clinical protocol, including the autonomous use of the IMU-based device. Figure 3 shows an example of tracing (participant #11). This patient wore the device from 11:45 a.m. to 7.00 a.m. of the next day, with an hour break from 4 p.m. to 5 p.m. due to a medical examination. According to the activity diary, the participant went to bed around 11:21 p.m. and during the first part of the night used mechanical ventilation. Participant #11 removed mechanical ventilation around 3 a.m. It can be noted that the patient’s respiratory rate during daily activities was maintained in the range of frequencies evaluated during tests performed in static conditions with the OEP (colored bands in Figure 3). It can also be noted that when mechanical ventilation was removed (around 4:00 a.m.), the respiratory rate became highly irregular.

### 3.5. Usability and Acceptability

**Figure 4:**
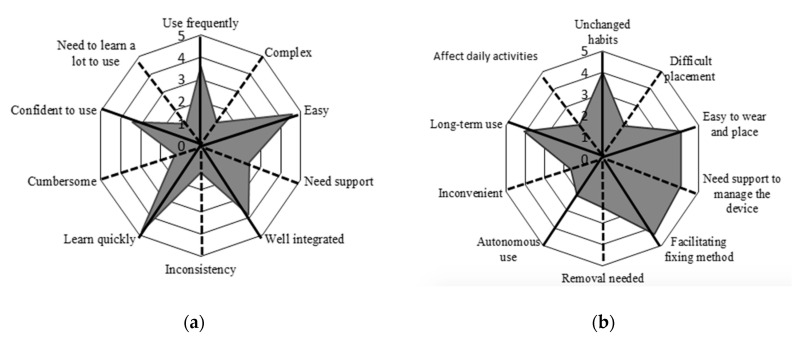
Radar plot of (**a**) the System Usability Scale (SUS) questionnaire, (**b**) ad-hoc questionnaire. The items and related mean scores of the questionnaire are synthetically reported (e.g., “Use frequently” corresponds to item #1 of the SUS “I think that I would like to use this system frequently”, “Unchanged habits” corresponds to item #1 of the ad-hoc questionnaire “It is possible to use the device without the need to modify my habits”; for a complete description of the items see Appendix A). The black solid lines indicate the items with a positive meaning, and black dotted lines indicate the items with a negative meaning. To obtain a compressive high score for both of the questionnaires we must obtain high scores for the positive items and low scores for the negative items.

All patients who participated in Phase B were asked to fill in questionnaires to evaluate usability and wearability of the device (SUS and ad-hoc questionnaires). Scores are shown in Figure 4.

Results are presented using radar plots that underline the mean scores obtained for each item of the questionnaires. Regarding the SUS questionnaire, the average score is 81.96 ± 12.98, associated with excellent usability according to the rating scales proposed by Bangor et al. [45]. The average score obtained from the ad-hoc questionnaire is 66.00 ± 17.06. Given the ad-hoc nature of this questionnaire, there is no available literature to evaluate the mean score; thus, we considered the scores item-by-item. Most participants reported that the device was easy to place and wear, the fixation method was comfortable, and that they would wear it for long periods of time. In contrast, one participant considered the device difficult to use autonomously, requiring the help of a caregiver, especially for the operations of placement and removal of the device.

### 3.6. Breathing Frequency: A Potential Marker of Respiratory Dysfunction

**Figure 5:**
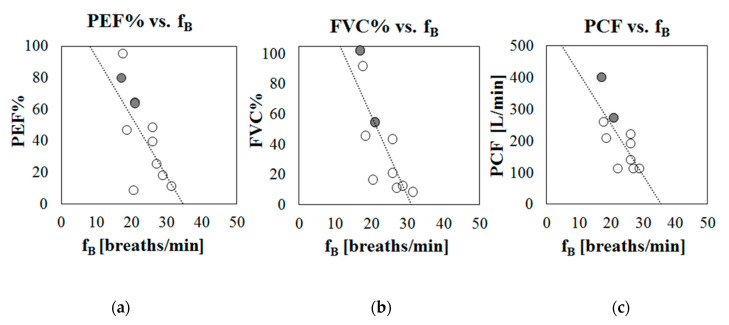
Scatter plots representing mean breathing frequency recorded during daily hours (Phase B) against indexes of respiratory function, for each participant. (**a**) % Peak Expiratory Flow with respect to predicted (PEF%), (**b**) % Forced Vital Capacity with respect to predicted (FVC%), (**c**) Peak Cough Flow (PCF) in liters per minute. Patients with LGMD2 are marked in grey.

To preliminarily investigate the potential of breathing frequency to predict respiratory dysfunction, the tracings of fB variation obtained during the daily use of the IMU-based device (Phase B of the protocol) were analyzed, both during day and night hours. For all of the participants that participated in Phase B of the protocol, periods of at least 12 min in which the subjects were seated on their wheelchair and not performing particular activities (eating, being examined by a clinician, talking, etc.) were selected and the mean breathing frequency was computed. Correlation analysis between mean breathing frequency at rest (during day hours) and age demonstrates that the level of breathing rate is not dependent on the age of the subject (Pearson correlation r = 0.013, *p* = 0.68). On the contrary, it was found that that breathing frequency was related to the respiratory function: breathing frequency at rest was negatively but significantly correlated with the indexes of respiratory function (PEF%: r = −0.71, *p* = 0.020; FVC%: r = −0.80, *p* = 0.005; PCF: r = −0.75, *p* = 0.013), meaning that higher breathing frequencies at rest are associated with worse respiratory functions. Figure 5 shows scatter plots in which the mean breathing frequencies recorded during day hours (Phase B) are plotted against the main indexes of respiratory function: Figure 5a peak expiratory flow (PEF% predicted); Figure 5b Forced Vital Capacity (FVC% predicted), and Figure 5c Peak Cough Flow (PCF in L/min).

## 4. Discussion

Careful, periodic assessment of respiratory function is crucial in patients with neuromuscular disease and, more generally, in patients at high risk of developing respiratory dysfunction and failure. In Duchenne Muscular Dystrophy, for example, major reported causes of death are respiratory insufficiency and heart failure, and respiratory management has the most important impact on survival. In these patients, a continuous monitoring of respiratory function, even if limited to a simple parameter such as breathing frequency, could help to follow the progression of the disease and to plan follow-up visits with increased awareness.

In this pilot study, a preliminary validation of a wearable, non-intrusive, IMU-based device for continuous breathing rate monitoring was carried out on a group of patients with neuromuscular disease. Compared to other wearable systems based on resistive, inductive, capacitive, and fiber optic sensors embedded in belts or shirts, IMU-based devices such as the one proposed in this study have several advantages. They are smaller and less intrusive and cumbersome, and can be positioned on several points of the thoraco-abdominal surface.

The aims of this pilot study were (1) to assess the ability of the device to accurately estimate breathing parameters in patients presenting shallow breathing, in a static condition; (2) verify the feasibility of use for long periods during daily life activities; (3) investigate usability and acceptability; and (4) preliminarily assess the possibility of using breathing frequency as a marker of respiratory dysfunction.

Regarding validation in static conditions, the measurements of breathing parameters obtained using the IMU-based device were compared with those obtained with Optoelectronic Plethysmography. The challenge in this case was to detect shallow breaths characterizing the breathing pattern typical of subjects with muscular weakness using the proposed system. The comparison between the measurements of fB obtained using the IMU-based device and using OEP provided optimal results, in terms of accuracy errors, correlation, and agreement. Regarding timing estimation (inspiratory and expiratory times), evidence was similar to those found in healthy subjects with the same device [27,28], i.e., reliability of the estimation was lower than that obtained for breathing frequency. However, correlation with measurements obtained using OEP was nonetheless relevant and significant.

The analysis of data recorded during the autonomous daily use of the device highlighted, initially, that the proposed device in its current form is able to acquire data for long periods, up to ~15 consecutive hours. The main concern regarded unexpected interruptions of acquisition data due to both data transmission issues and intrinsic protocol inefficiency. Nevertheless, the efficiency in terms of data analysis, defined as the number of files from which it was possible to extract a mean breathing frequency with respect to the total number of recorded files, was high (~85%). An example case was presented in detail showing the whole tracing of breathing frequency variation recorded with the device for a total period of 20 h, during day and night hours. Using the same processing algorithm previously presented and used for healthy subjects [27], it was possible to recover breathing frequency for most of the dataset, including dynamic conditions and challenging situations, including irregular breathing due to concomitant activities, such as eating and speaking. Nevertheless, in these cases, supervision of an operator was needed during data analysis, contrary to the case for static conditions (completely automatic algorithm).

Regarding usability and acceptability of the proposed system, participants positively evaluated the device for ease of use, comfort, usability, and wearability, as recorded in the SUS and the ad-hoc questionnaire. The SUS questionnaire obtained an overall mean score of approximately 82, indicating excellent usability [45]. Moreover, preliminary results confirmed that breathing frequency is a valuable breathing parameter to monitor, at the clinic and at home, because it strongly correlates with the main indexes of respiratory function (PEF%, FVC%, and PCF).

To the best of our knowledge, this is the first time that a system based on inertial sensors has been used to record breathing frequency and temporal parameters in patients with neuromuscular disorders, and this constitutes a strength of the present work. Moreover, the tests undertaken in this study did not only consider the validation of the system in a clinical population in terms of accuracy, which is an original aspect per se, but also the assessment of the feasibility of the proposed device for prolonged monitoring during daily activities. This involved investigation of patients’ perception of acceptance, usability, and comfort of the device. This is highly important for the process of technology transfer to clinical practice, because studies in the literature involving validation of this kind of system on clinical populations are rare and of a preliminary nature [22,54,55].

A weakness of this study is the absence of a reference system for daily use, which limits the conclusions that can be drawn in terms of accuracy of the estimation in dynamic conditions, and thus leaving room for qualitative speculations only. This is also due to the fact that a validated method for non-intrusive breathing rate assessment in dynamic conditions and during daily activities is not available. However, the aim of the pilot study was to firstly assess feasibility, wearability, and usability to collect useful information, suggestions, and data for further improvements of the device. Once the necessary adjustments emerging from the pilot study are implemented, an extended validation study should be performed, with a larger sample size and including a comparison with a validated, intrusive reference measurement method (such as flowmeters and metabolic charts). This might define a limitation for the assessed activities under dynamic conditions that can be evaluated in a laboratory (such as speech and wheelchair movement). Another limitation is that analysis of data acquired during long-term monitoring is operator dependent and not completely automatic. In future studies, the processing algorithm can be further improved, taking advantage of the presence of the reference unit, including automatic classification of static and non-static periods, and identification of the level and kind of activity using, for example, machine learning classification algorithms. The extraction of breathing parameters for non-static periods may be achieved by adapting the algorithm to the level of activity, and changing key parameters and thresholds that are constant for the static condition analysis, such as the smoothing degree and frame length used for baseline removal. IN addition, a set of rules to automatically identify and exclude non-reliable values in the breathing rate variation must be implemented. These improvements, together with the refinement of the mobile app and server, will lead to a complete platform for tele-monitoring of breathing pattern during daily life activities.

The results obtained in this pilot study will allow improvement of the device in terms of design (e.g., housing shape, fixation methods, on/off management) and processing algorithm optimization. The device proposed in this work represents a step forward for the implementation of at-home continuous respiratory function monitoring in patients at high risk of developing respiratory dysfunction and failure. In the future, a study investigating the capability of the system for detecting and characterizing thoraco-abdominal asynchronies will be conducted, fully exploiting the potential of the modularity of the device. Moreover, improvement of the analysis algorithm allowing on-line extraction of the breathing parameters, and automatic unsupervised analysis during daily life activities, will foster the use of the device in other applications, such as sport and fitness, exercise testing, rehabilitation protocols, and treatment evaluation, in which respiratory assessment could be of significant interest.

## 5. Patents

The present work is partially described in the International Patent application n° PCT/IB2018/054956, priority date 11 July 2017, title “A wearable device for the continuous monitoring of the respiratory rate”. Inventors: Ambra Cesareo, Andrea Aliverti, Assignee: Politecnico di Milano.

## Figures and Tables

**Table 1:** Anthropometric and clinical characteristics of the participants.

Parameter		DMD (n = 13)	LGMD (n = 2)
Age (years)		23.99 ± 5.94	53 ± 0.04
Weight (kg)		54.77 ± 14.96	86 ± 45.96
Height (cm)		166 ± 10	180 ± 7
BMI (km∗m^−2^)		19.77 ± 4.64	26.41 ± 11.41
Gene mutation (N)	Deletion	9	-
	Duplication	1	-
	Point mutation	3	-
Scoliosis (N)	No	0	1
	Mild	1	1
	Moderate	2	0
	Severe	7	0
	Spinal fusion	2	0
NIV (N)		10	1
Heart dysfunction (N)		12	1
Use of M-IE (N)		11	0

N = 15 subjects. Data are presented as N subjects or mean ± SD. NIV: noninvasive ventilation; MI-E: Mechanical In-exsufflator (cough assist device).

**Table 2:** Respiratory Function.

	Parameter	DMD (n = 13)	LGMD (n = 2)
**Spirometry**	FVC [L]	1.18 ± 0.90	3.45 ± 0.99
	FVC (% pred)	28.44 ± 27.34	78.00 ± 33.94
	FEV1 [L]	1.10 ± 0.87	2.65 ± 0.85
	FEV1 (% pred)	31.44 ± 31.87	75 ± 33.94
	FEF25–75% [L/sec]	1.68 ± 1.37	2.32 ± 1.12
	FEF 25–75% (% pred)	35.11 ± 29.19	61.00 ± 32.53
	FEF50 [L/sec]	2.07 ± 1.63	3.71 ± 1.14
	FEF50 (% pred)	43.67 ± 39.70	79.00 ± 31.11
	PEF [L/sec]	2.75 ± 1.72	6.33 ± 0.46
	PEF (% pred)	33.44 ± 27.74	72.00 ± 11.31
**Lung volumes**	TLC [L]	4.52 ± 1.42	6.41 ± 0.36
	TLC (% pred)	77.40 ± 25.91	90 ± 7.07
	RV [L]	2.80 ± 1.10	2.96 ± 1.35
	RV (% pred)	189.80 ± 52.45	128.5 ± 50.20
	FRC [L]	3.54 ± 1.22	4.26 ± 0.35
	FRC (% pred)	120.63 ± 36.55	119.5 ± 0.71

Data are expressed as mean ± SD for all of the participants divided by the type of muscular dystrophy (DMD, n = 13; LGMD, n = 2). FEF25–75%: forced expiratory flow during the middle half of the FVC maneuver; FEF50: instantaneous flow at the moment the subject has exhaled 50% of FVC; PEF: peak expiratory flow; TLC: total lung capacity; FRC: functional residual capacity; RV: Residual volume.

**Table 3:** Absolute and relative estimation errors.

Parameter	Position	Compartment	E	E%
**f_B_**	Supine	thorax	0.79 (0.55; 1.11)	4.53 (2.86; 6.17)
		abdomen	1.08 (0.53; 1.56)	3.53 (1.61; 8.29)
	Seated	thorax	1.09 (0.73; 0.73)	4.99 (3.11; 0.48)
		abdomen	1.02 (0.60; 1.50)	5.31 (3.25; 7.06)
**T_I_**	Supine	thorax	0.24 (0.37; 0.13)	16.82 (25.43; 9.03)
		abdomen	0.279 (0.42; 0.14)	20.67 (30.15; 9.61)
	Seated	thorax	0.149 (0.36; 0.08)	10.79 (24.48; 6.46)
		abdomen	0.349 (0.50; 0.10)	24.29 (40.30; 7.96)
**T_E_**	Supine	thorax	0.19 (0.32;0.09)	11.24 (15.10; 5.15)
		abdomen	0.20 (0.20;0.19)	11.13 (22.84; 6.90)
	Seated	thorax	0.11 (0.26;0.04)	6.89 (15.60; 2.85)
		abdomen	0.19 (0.34; 0.13)	13.21 (22.01; 8.96)

Data are expressed as median and interquartile range (75th percentile; 25th percentile) for all of the subjects and trials in supine and seated position, for thoracic and abdominal compartments. Absolute errors (E) are expressed in breaths/minute for f**_B_**, and in seconds for T_I_ and T**_E_**.

## Appendix A

The ad hoc questionnaire was administered at the end of Phase B to collect feedback from participants about acceptance and wearability of the device. This scale was designed with 10 items phrased positively or negatively:

1. It is possible to use the device without the need to modify my habits.
2. It was difficult to learn using the device.
3. I think the device is easy to wear and place.
4. I think I would need someone to help me managing the device.
5. The fixation method of the device units facilitates the placement and improve the wearability.
6. Sometimes I preferred to remove the device for a period.
7. I think I would be able to use the device autonomously (placement, activation, app management, etc.…).
8. I found the fixation method uncomfortable.
9. I think I could wear the device for a long period.
10. I think that the use of the device would negatively affect my daily activities.

The score of this scale is computed as described for the SUS: it ranges from 0 to 100, with higher values (>50) meaning high perceived usability and wearability of the system.
